# Silencing of Hypoglossal Motoneurons Leads to Sleep Disordered Breathing in Lean Mice

**DOI:** 10.3389/fneur.2018.00962

**Published:** 2018-11-14

**Authors:** Thomaz A. Fleury Curado, Huy Pho, Olga Dergacheva, Slava Berger, Rachel Lee, Carla Freire, Aya Asherov, Luis U. Sennes, David Mendelowitz, Alan R. Schwartz, Vsevolod Y. Polotsky

**Affiliations:** ^1^Division of Pulmonary and Critical Care Medicine, Department of Medicine, Johns Hopkins University School of Medicine, Baltimore, MD, United States; ^2^Department of Otolaryngology, University of Sao Paulo, São Paulo, Brazil; ^3^Department of Pharmacology and Physiology, The George Washington University, Washington, DC, United States

**Keywords:** obstructive sleeep apnea, chemogenetic, sleep, upper airway, neuromuscular activity

## Abstract

Obstructive Sleep Apnea (OSA) is a prevalent condition and a major cause of morbidity and mortality in Western Society. The loss of motor input to the tongue and specifically to the genioglossus muscle during sleep is associated with pharyngeal collapsibility and the development of OSA. We applied a novel chemogenetic method to develop a mouse model of sleep disordered breathing Our goal was to reversibly silence neuromotor input to the genioglossal muscle using an adeno-associated viral vector carrying inhibitory designer receptors exclusively activated by designer drugs AAV5-hM4Di-mCherry (DREADD), which was delivered bilaterally to the hypoglossal nucleus in fifteen C57BL/6J mice. In the *in vivo* experiment, 4 weeks after the viral administration mice were injected with a DREADD ligand clozapine-N-oxide (CNO, i.p., 1mg/kg) or saline followed by a sleep study; a week later treatments were alternated and a second sleep study was performed. Inspiratory flow limitation was recognized by the presence of a plateau in mid-respiratory flow; oxyhemoglobin desaturations were defined as desaturations >4% from baseline. In the *in vitro* electrophysiology experiment, four males and three females of 5 days of age were used. Sixteen–nineteen days after DREADD injection brain slices of medulla were prepared and individual hypoglossal motoneurons were recorded before and after CNO application. Positive mCherry staining was detected in the hypoglossal nucleus in all mice confirming successful targeting. In sleep studies, CNO markedly increased the frequency of flow limitation n NREM sleep (from 1.9 ± 1.3% after vehicle injection to 14.2 ± 3.4% after CNO, *p* < 0.05) and REM sleep (from 22.3% ± 4.1% to 30.9 ± 4.6%, respectively, *p* < 0.05) compared to saline treatment, but there was no significant oxyhemoglobin desaturation or sleep fragmentation. Electrophysiology recording in brain slices showed that CNO inhibited firing frequency of DREADD-containing hypoglossal motoneurons. We conclude that chemogenetic approach allows to silence hypoglossal motoneurons in mice, which leads to sleep disordered breathing manifested by inspiratory flow limitation during NREM and REM sleep without oxyhemoglobin desaturation or sleep fragmentation. Other co-morbid factors, such as compromised upper airway anatomy, may be needed to achieve recurrent pharyngeal obstruction observed in OSA.

## Introduction

Obstructive sleep apnea (OSA) is due to recurrent closure of upper airway during sleep leading to intermittent hypoxia and fragmentation of sleep (1). OSA is a common disease affecting ≥30% of the adult population (2). OSA predisposes to motor vehicle and industrial accidents and contributes significantly to the development and progression of neurocognitive, metabolic, cardiovascular, and oncologic diseases (2–7). Nasal continuous positive airway pressure (CPAP) relieves upper airway obstruction during sleep but does not reverse underlying defects in pharyngeal collapsibility. Poor adherence to CPAP severely limits its use, and few effective alternatives to CPAP exist (8). There is no effective pharmacotherapy for OSA (9–11). Improved understanding of the disease pathogenesis is a key determinant of successful drug development (12). The creation of an adequate rodent model that mimics human OSA would facilitate the progress in the field.

Anatomic predisposition plays a primary role in OSA pathogenesis (11), whereas faulty neuromuscular mechanisms during sleep fail to compensate adequately for compromised pharyngeal patency (13–16). Neuromuscular compensatory responses are particularly important in stabilizing the upper airway during sleep. The tongue plays a critical role in the pathogenesis of OSA and dysfunction of the principal pharyngeal dilator, genioglossus (GG) muscle, during sleep contributes to the OSA pathogenesis (12, 16–19). Augmenting tongue muscle activity with hypoglossal nerve stimulation improves airway patency and treats OSA (20, 21). In this study we explored whether inhibition of hypoglossal motoneurons may lead to sleep disordered breathing even in the absence of the compromised upper airway anatomy.

There are a number of differences in the upper airway anatomy between mice and humans (22). Nevertheless, we have repeatedly demonstrated that that upper airway function and treatment responses to stimulation of the hypoglossal nerve and of lingual protrudor and retractor muscles are comparable between mice and humans [(16, 23–28)]. We have developed complementary mouse models of upper airway obstruction (25, 29) and OSA (23, 26, 30). Moreover, we have demonstrated that obesity plays a major role in the pathogenesis of sleep disordered breathing in mice and humans alike (14, 23, 24, 26).

Similarities in hypoglossal nerve and lingual muscle anatomy and upper airway physiology between mice and men led us to a concept of selective targeting of hypoglossal motoneurons using a chemogenetic approach. The principal tools of chemogenetics are designer receptors exclusively activated by designer drugs (DREADDs). DREADDs are G-coupled receptors genetically modified to recognize unique and otherwise inert ligands. DREADDS are widely used in neuroscience to remotely control cellular signaling, neuronal activity, and behavior (31). We have previously used stereotactic brain injections and deployed an adeno-associated viral vector harboring a mutated excitatory human acetylcholine muscarinic receptor subtype 3 (rAAV5-hSyn-hM3(Gq)-mCherry in the hypoglossal nucleus of C57BL/6J mice (32). A DREADD ligand clozapine-N-oxide (CNO) increased GG tonic and phasic activity and, most importantly, markedly dilated the pharynx on dynamic MR images in anesthetized mice (32). Other investigators used a similar approach to show that chemogenetic stimulation of hypoglossal motoneurons increases GG muscle activity across sleep/wake stages (33). These reports suggest that stimulation of hypoglossal motoneurons carrying excitatory DREADDs may alleviate OSA.

In the current study, we examined whether chemogenetic inhibition of hypoglossal motoneurons would lead to sleep disordered breathing in lean C57BL/6J mice. We used inhibitory DREADDs expressing the mutated human muscarinic receptor hM4(Gi). Stimulation of this receptor with CNO activates G-protein inwardly rectifying potassium (GIRK) channels thereby hyperpolarizing and silencing neuronal activity (31). Inhibitory DREADDs were deployed in the hypoglossal motoneurons by stereotactic brain injection and then sleep studies were performed after CNO or saline administration. The tongue is a complex structure which consists of extrinsic and intrinsic muscles (34). Extrinsic muscles determine tongue movements, whereas intrinsic muscles determine the shape of the tongue. Extrinsic muscles are subdivided in tongue protrudors and retractors. Inhibition of hypoglossal motoneurons innervating the main tongue protrudor and pharyngeal dilator, genioglossus muscle, may lead to upper airway obstruction, and OSA (34). Although exclusive targeting of the hypoglossal motoneurons is not technically positive, we aimed our stereotactic injections at the ventral rostral portion of the 12 nucleus, which has greater representation of genioglossus innervation (35, 36). In parallel, electrophysiological silencing of these neurons by CNO was confirmed in brain slices. The overarching goal of the study was to create a novel rodent model of sleep disordered breathing.

## Methods

### Animals

In total, fifteen C57BL/6J mice of both sexes from Jackson Laboratories, Bar Harbor, ME were used in all experiments. Eight male mice were used for *in vivo* experiment. These mice were 10 weeks of age and 28.0 ± 0.4 g of weight at the beginning of the experiment, were fed with a chow diet and free water *ad libitum* and housed in a 22°C laboratory with a 12 h light/dark cycle (light phase 9am−9pm). The study complied with the American Physiological Society Guidelines for Animal Studies and was approved by the Johns Hopkins University Animal Use and Care Committee. Mice were used for experiments with viral vectors for DREADD expression and treated with the DREADD ligand clozapine-N-oxide (CNO) and saline in crossover fashion.

For electrophysiology studies, experiments were conducted in seven C57/BL/6J mice of both sexes (four males and three females). At the time of DREADD injection mice were 5 days old. All animal procedures were performed in compliance with the institutional guidelines at George Washington University and are in accordance with the recommendations of the Panel on Euthanasia of the American Veterinary Medical Association and the National Institutes of Health Guide for the Care and Use of Laboratory Animals.

## *In vivo* experiments

### Viral vector administration

For the *in vivo* experiment, mice were anesthetized with isoflurane for induction (2–3% in closed chamber) and placed in the Kopf stereotaxic apparatus (model 963; Kopf Instruments, Tujunga, CA, USA) with mouse adapter VetEquip, and isoflurane vaporizer. Anesthesia was subsequently maintained at 1–2% isoflurane. DREADD (rAAV5-hSyn-hM4(Gi)-mCherry, Vector Core at the University of North Carolina at Chapel Hill (UNC Vector Core), 7 × 1012 vg per ml, 40 nl) were delivered bilaterally using pre-pulled glass micropipettes (Sutter, Novato, California) to the rostral portion of the hypoglossal nucleus of the medulla using the following stereotactic coordinates from the animal's bregma: 7.20 mm anterior-posterior, 0.20 mm medial-lateral bilateral and 4.75 mm dorso-ventral. The stereotactic coordinates were determined based on The Mouse Brain Atlas.

### Electroencephalogram/ electromyogram electrode implantation

Electroencephalogram (EEG) and electromyogram (EMG) electrodes were implanted with an EEG/EMG Headmount (Pinnacle Technology, Lawrence, KS) as previously described (25). Briefly, four electroencephalographic (EEG) Teflon-coated stainless steel wire electrodes were inserted into the skull through predrilled holes and bonded to the dorsal surface of the skull with dental acrylic (Land Dental, Wheeling, IL) under 1–2% isoflurane anesthesia. Two nuchal electromyographic (EMG) electrodes were tunneled subcutaneously and placed over the nuchal muscles posterior to the skull. Mice were allowed to recover for 3–4 days prior to polysomnography.

### Experimental design and mouse polysomnography

Four–Six weeks after DREADD administration, mice were injected with CNO at 1 mg/kg (*n* = 4) or saline (*n* = 4) intraperitoneally followed by sleep recording. A week later animals were crossed over and CNO-treated animals were given saline whereas, saline-treated animals received CNO.

Whole body plethysmography (WBP) recordings (mouse whole body plethysmograph, Buxco, Wilmington, NC) were performed as previously described (37). In brief, the animals were placed in a modified WBP open-system chamber to allow for prolonged ~6 h recordings (10:00 am−04:00 pm). The WBP chamber had the internal diameter of 80 mm, the height of 50.5 mm and the volume ~0.4 L. The chamber was equipped with two ports (pneumotachographs) on the upper surface and one large-side port and three small-side ports at the base, which we utilized to customize our system and optimize its performance. Positive and negative pressure sources were utilized in series with mass flow controllers and high-resistance elements to generate a continuous bias flow through the animal chamber while maintaining a sufficiently high time constant. The reference chamber serves to filter ambient noise from the pressure signal. Slow leaks present on both chambers allowed for equilibration with atmospheric pressure. A respiratory effort sensor bladder was placed under the mouse to transduce the mechanical pressure changes associated with mouse breathing, while the reference bladder signal allowed for cancellation of the contaminating chamber pressure signal via the differential pressure transducer. The Drorbaugh and Fenn equation was used to calculate the WBP tidal volume (VT) signal from the WBP chamber pressure signal. Application of this formula required the measurement of the following variables during each WBP recording session: mouse rectal temperature, chamber temperature, room temperature and relative humidity, and chamber gas constant, which was calculated by utilizing a known volume injection and the resultant WBP pressure deflection.

Mice were habituated to the WBP chamber from 10:00 am to 04:00 pm 1 day before the recording session. During full polysomnographic recording sessions, the chamber was humidified to 90% relative humidity, and the mouse was allowed 45 min to acclimate to the chamber before recording. All signals were digitized at 1,000 Hz (sampling frequency per channel) and recorded in LabChart 7 Pro (Version 7.2, ADInstruments, Dunedin, NZ). Respiratory signals were analyzed from all REM sleep periods and from periods of NREM sleep sampled periodically at 20 s stretches every half an hour throughout the total recording time. NREM stretches were selected from NREM periods of at least 1 min. Custom software was used to demarcate the start and end of inspiration and expiration for subsequent calculations of timing and amplitude parameters for each respiratory cycle. Oxyhemoglobin saturation (SpO_2_) was monitored using pulse oximetry with a mouse neck collar clip (Starr Life Science, Oakmont, PA). Body temperature was measured by a rectal probe. Additional customized software was used to demarcate each inspiration, classify the breath as flow limited and describe maximal inspiratory airflow (V_I_max) and components of minute ventilation (V_E_).

### Sleep and respiratory analysis

Studies were scored by two independent scorers (RL and HP), who were blinded to study conditions. Sleep-wake state was scored visually in 5 s epochs from 10:00 am until 04:00 pm. Standard criteria were employed to score sleep-wake state based on EEG and EMG frequency content and amplitude, as previously described (23). Wakefulness was characterized by low-amplitude, high-frequency (~10 to 20 Hz) EEG waves and high levels of EMG activity compared with the sleep states. NREM sleep was characterized by high-amplitude, low frequency (~2 to 5 Hz) EEG waves with EMG activity considerably less than during wakefulness. REM sleep was characterized by low-amplitude, mixed frequency (~5 to 10 Hz) EEG waves with EMG amplitude either below or equal to that during NREM sleep.

The instantaneous respiratory rate (RR, breaths/min) was calculated as the reciprocal of the respiratory period, and the instantaneous V_E_ (mL/min) was product of the RR and V_T_ for each breath. SpO_2_ was monitored using neck collar clip pulse oximetry. The oxygen desaturation index (ODI) was defined as ≥4% oxyhemoglobin desaturation from the baseline (ODI) for at least two breaths. T90 was defined as the percentage of total sleep time spent with SpO_2_ <90%.

We utilized the airflow and respiratory effort signals to develop an algorithm for detecting upper airway obstruction during sleep. Obstruction was characterized by the development of inspiratory flow limitation (IFL) in the presence of increased effort. Custom software provided a discrete value for inspiratory flow (Vi) at the inspiratory midpoint (Vi50). This midpoint was then used to partition inspiration into early and late phases. Peak inspiratory flow during the early and late phases were defined as Vimax1 and Vimax2, respectively. Breaths were considered to be non-flow limited when a plateau in mid-inspiratory flow could not be discerned. Sniffs were defined by their short duration when the total inspiratory time was <1.75^*^SD of the mean inspiratory time of the entire sample of breaths, and were excluded. For the remaining inspirations with durations >1.75^*^SD, IFL was defined if a plateau in mid-inspiratory flow was detected, as follows. (1) Breaths with an early peak of inspiratory flow had to be followed by a plateau of sufficient duration. A plateau was considered to be present if Vimax1 was greater than both Vi50 and Vimax2 (decrescendo pattern), and if the time between the early and late flow peaks were ≥20% of the total inspiratory time. At times, a decrease in flow could be discerned beyond the point of Vimax1, consistent with the phenomenon of negative effort dependence in IFL breaths. (2) Alternatively, IFL was defined for breaths in which the late inspiratory peak in airflow exceeded the early peak. Once breaths were deemed to be flow-limited, we defined the severity of airflow obstruction by the absolute peak level of Vimax.

### Histology

Mice were sacrificed by isoflurane overdose and rapidly perfused with phosphate buffered saline (PBS). The brains were carefully removed, cryoprotected in 20% sucrose in PBS overnight at 4°C. The next morning, brains were frozen on dry ice and stored in antifreeze solution at −20°C until further use. A subset of mice (*n* = 5) was injected with a retrograde fluorescent tracer cholera toxin B conjugated to Alexa Fluor™ 488 (CTB-AF488, ThermoFischer, Waltham MA) bilaterally 5 μl per site x 2 (10 μl in total) under 1–2% isoflurane anesthesia. The injections were performed into the dorso-caudal segment of the tongue posterior to the intermolar eminence by the dorsal approach, 0.5 mm laterally to the midline using a 10 μ Hamilton syringe. The depth of the injection was 1 mm as assured by the needle cuff. Previous studies in rodents demonstrated that injections according to these coordinates target predominantly extrinsic muscles of the tongue, specifically, the genioglossus muscle (35, 36). Mice were sacrificed 72 hs later. The medulla was cut into 10-μm-thick coronal sections on a sliding microtome (Thermo Scientific HM 560; Waltham, MA, USA). The sections were performed via the entire medulla. mCherry and AF488 fluorescence was visualized with the Olympus BX41 microscope with the Image Pro Plus analysis software (Media Cybernetics).

### Slice preparation and electrophysiology

For the *in vitro* recording mouse pups were anesthetized with hypothermia. Animals were mounted in a stereotactic apparatus with a neonatal adapter (Stoelting, Wood Dale, IL). The skull was exposed, and a small hole was made to position a pulled calibrated pipette (VWR, Radnor, PA) containing viral vector. Viral vector (20 nl) was slowly injected at the following stereotactic coordinates from the bregma: −4.2 mm anterior-posterior, 0 mm medial-lateral and 1.6 mm dorso-ventral. After surgery, buprenorphine was administered, and animals were monitored for 30 min and every 20 min thereafter until ambulatory.

On the day of experiment, animals (21–24 days old) were anesthetized with isoflurane, sacrificed and glycerol-based artificial cerebrospinal fluid (aCSF) was perfused transcardially. Glycerol-based aCSF (4°C) contained (in mM): 252 glycerol, 1.6 KCl, 1.2 NaH2PO4, 1.2 MgCl, 2.4 CaCl2, 26 NaHCO3, and 11 glucose. The brain was removed, and 300-μm-thick coronal slices of the medulla containing the hypoglossal nucleus were obtained with a vibratome. The slices were then transferred to a solution of the following composition (in mM) 110 N-methyl-d-glucamine (NMDG), 2.5 KCl, 1.2 NaH2PO4, 25 NaHCO3, 25 glucose, 0.5 CaCl2, and 10 mM MgSO4 equilibrated with 95% O2–5% CO2 (pH 7.4) at 34°C for 15 min. The slices were then transferred from NMDG-based aCSF to a recording chamber, which allowed perfusion (5–10 ml/min) of aCSF at room temperature (25°C) containing (in mM) 125 NaCl, 3 KCl, 2 CaCl2, 26 NaHCO3, 5 glucose, and 5 HEPES equilibrated with 95% O2–5% CO2 (pH 7.4) for at least 30 min before experiments were conducted.

Individual neurons in the hypoglossal nucleus were identified by the presence of the fluorescent tracer. These identified neurons were then imaged with differential interference contrast optics, infrared illumination, and infrared-sensitive video detection cameras. Patched pipettes (2.5–3.5 MΩ) were filled with a solution consisting of 135 mM K-gluconic acid, 10 mM HEPES, 10 mM EGTA, 1 mM CaCl2, and 1 mM MgCl2. Action potentials of hypoglossal neurons were recorded in current clamp configuration with an Axopatch 200B and pClamp 9 software (Axon Instruments). Clozapine-N-oxide (CNO, 10 μM) was applied to examine its effects on spontaneous action potential firing of DREADDs expressing hypoglossal neurons.

Analysis of firing activity of hypoglossal neurons was performed using Mini Analysis (version 5.6.12; Synaptosoft, Decatur, GA). Firing activity was analyzed from 2 min periods prior to CNO application (control), and in sequential 2-min periods for 20 min post CNO application. Results are presented as means ± SE and statistically compared by ANOVA with repeated-measures and Dunnett's posttest. Significant difference was set at *P* < 0.05.

### Statistical analysis

The primary objective of the present study was to examine effects of CNO in SDB. Our primary outcome was the percentage of flow-limited breaths during sleep. Our secondary outcomes were severity of disturbances in ventilation and oxygenation. The statistical analysis was designed to examine effects of CNO on measures of upper airway obstruction, ventilation, and gas exchange disturbances. Specifically, upper airway obstruction severity was characterized by the percentage of breaths that exhibited IFL and the level of VImax during flow limited breathing. Ventilation was assessed by VE and its components, VT and RR, during non-flow limited and flow limited breathing. The severity of gas exchange abnormalities was characterized by T90 and mean SpO2 across sleep/wake states. The periodicity of gas exchange abnormalities (measures of sleep apnea) was assessed by ODI. SDB parameters were compared between CNO and saline with the Wilcoxon rank-sum tests (STATA 12, StataCorp LP, College Station TX). Because sleep stage can markedly affect SDB severity, we stratified analyses by NREM vs. REM sleep. In all cases, a *p*-value < 0.05 was considered significant.

We powered our study to detect an increase in percentage of IFL breaths after CNO compared to saline of 10%, with a within-group standard deviation of 5%. Under these assumptions, we calculated a standardized effect size of 2. We, therefore, estimated that six mice (using animals as their own control, CNO vs. saline) would result in a 90% chance to detect a difference in the IFL prevalence.

## Results

**Figure 1:**
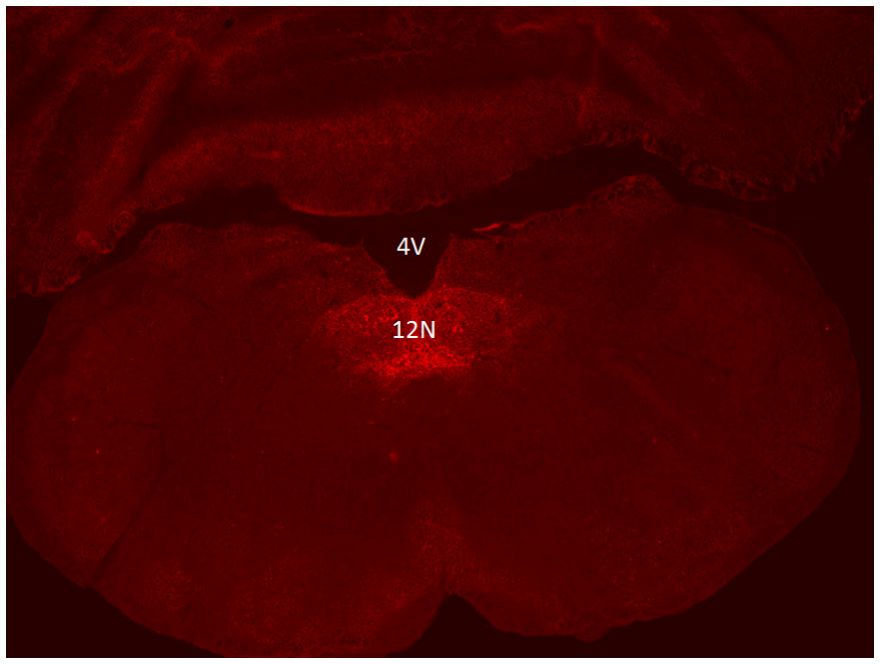
Localization of AAV5-hSyn-hM4 (Gi)-mCherry DREADD in the hypoglossal nucleus. Fluorescent microscopy images (× 10) show mCherry expression spanning the hypoglossal nucleus. 12N denotes the hypoglossal nucleus; 4V denotes the fourth ventricle.

**Figure 2:**
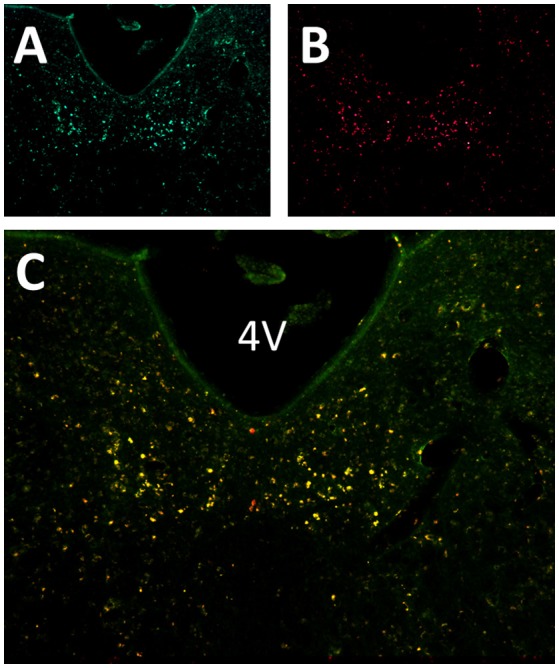
Localization of AAV5-hSyn-hM4 (Gi)-mCherry and fluorescently labeled cholera toxin B (CTB-AF488) in the hypoglossal nucleus. Fluorescent microscopy images (× 20) shows **(A)** CTB labeled motoneurons within the rostral hypoglossal nucleus following genioglossal injection; **(B)** mCherry expression in the same section; **(C)** merged mCherry and AF488 images. 4V denotes 4th ventricle.

**Figure 3:**
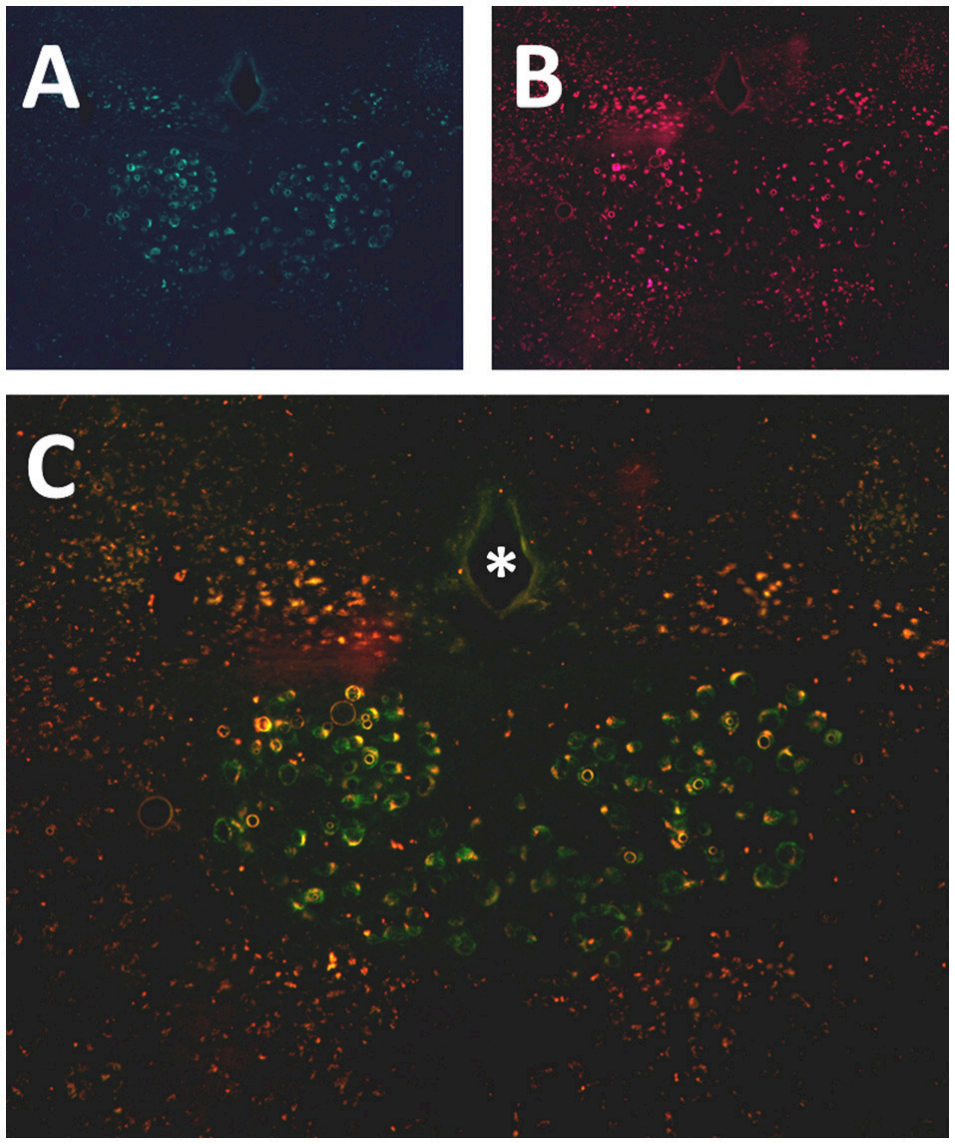
Localization of AAV5-hSyn-hM4 (Gi)-mCherry and fluorescently labeled cholera toxin B (CTB-AF488) in the hypoglossal nucleus. Fluorescent microscopy images (× 20) shows **(A)** CTB labeled motoneurons more caudally, at the obex level of the hypoglossal nucleus, following genioglossal injection; **(B)** mCherry expression in the same section; **(C)** merged mCherry and AF488 images. ^*^ denotes central canal.

Six to eight weeks after infection with rAAV5-hSyn-hM4(Gi), mCherry expression was localized to the hypoglossal nucleus in all eight mice (Figure 1). Five out of eight mice were injected with CTB-AF488 into the genioglossus muscle to confirm localization of DREADDs to hypoglossal motoneurons innervating the tongue protrudors. In the rostral portion of the 12 nucleus, where the genioglossus subcompartment of the hypoglossal nucleus is highly represented, especially ventrally (35, 36), a majority of motoneurons expressed DREADDs (Figure 2). All CTB expressing neurons colored in green (Figure 2A) changed to orange upon merging with mCherry DREADDs (Figures 2B,C). However, a few DREADD positive neurons did not change color from red to orange suggesting that these motoneurons innervated other lingual muscles. In contrast, caudally, in the obex region many mCherry positive neurons did not contain CTB, especially dorsally, indicating that retractors and may be intrinsic tongue muscles were also DREADD transfected (Figures 3A–C). Unlike the rostral portion of the 12 nucleus, the caudal portion contained CTB-positive DREADD-negative motoneurons, because our stereotactic injection was more rostral.

**Figure 4:**
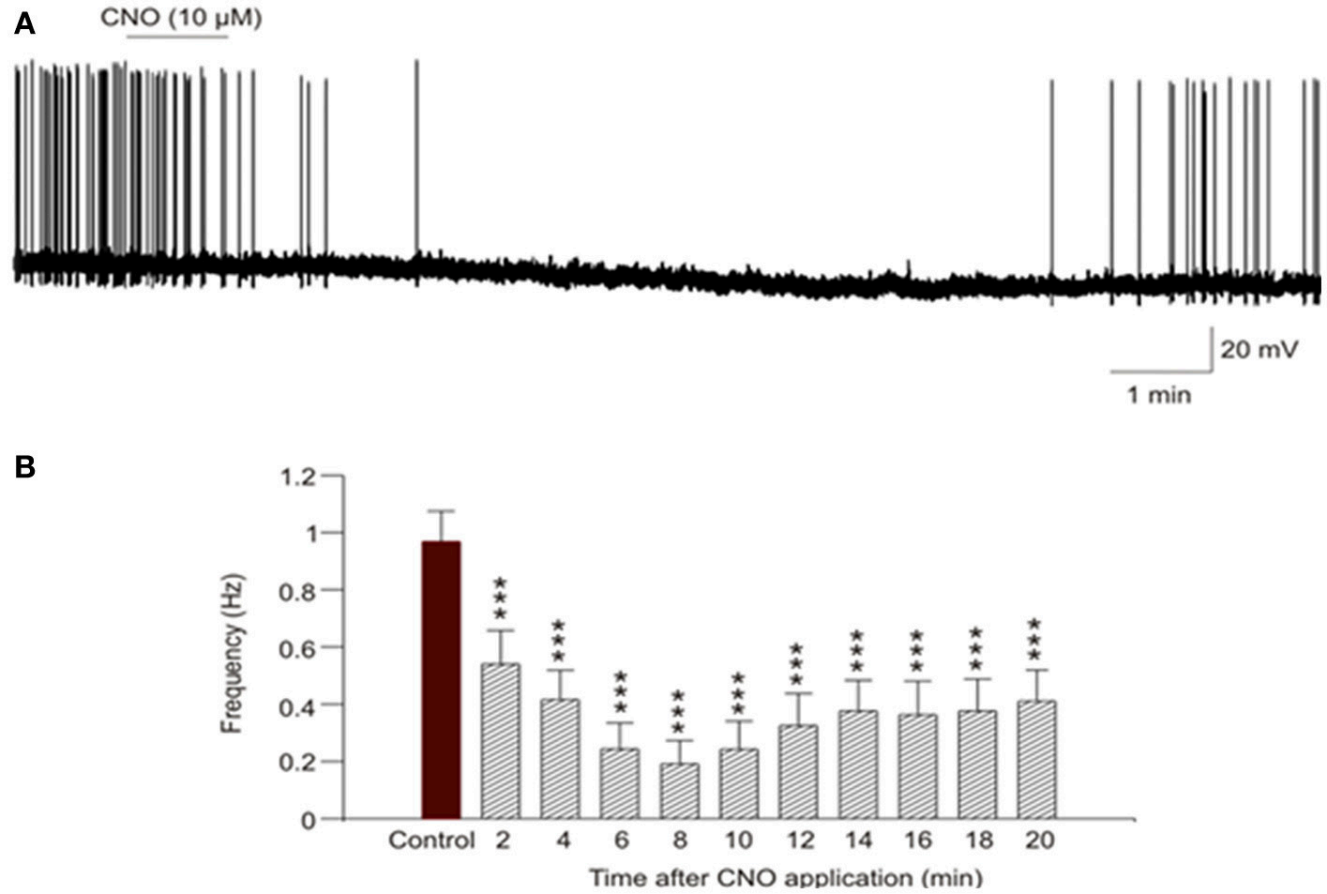
*In-vitro* activation of hypoglossal neurons that contain inhibitory DREADDs. **(A)** Representative example demonstrates a depression of action potential firing of DREADDs-containing hypoglossal neuron recorded in current-clamp configuration after 1-min CNO (10 μM) application. **(B)** The summary data from 7 neurons illustrate significant (*P* < 0.001, 1-way ANOVA) inhibition of firing activity of hypoglossal neurons that started as early as 2 min post CNO application and lasted for at least 20 min post CNO application. ^***^ *P* < 0.001.

The responses of individual hypoglossal neurons upon activation of hSyn-hM4(Gi)-mCherry were assessed using *in vitro* patch-clamp techniques. The action potential firing frequency of DREADDs-containing hypoglossal neurons was significantly inhibited within 2 min after CNO application (from 0.97 ± 0.11 Hz to 0.54 ± 0.12 Hz; *n* = 7; *P* < 0.001 Figure 4A). This inhibition of firing activity in hypoglossal neurons was maintained for at least 20 min post CNO application (0.97 ± 0.11 Hz, control vs. 0.41 ± 0.11 Hz, 20 min post CNO; *n* = 7; *P* < 0.001; 1-way ANOVA; Figure 4B.)

CNO activation of DREADDs in the *in-vivo* studies did not affect body weight or sleep architecture (Tables 1, 2). Non-flow limited breathing and flow limited breathing indexes characterize respiratory pump and upper airway function during sleep, respectively and therefore were analyzed separately. Inhibiting hypoglossal motoneuron activity had no effect on the parameters of non-flow limited breathing. There was no significant change in minute ventilation, tidal volume, respiratory rate or mean SpO_2_ in NREM or REM sleep (Table 3).

**Table 1:** Basic characteristics of C57BL/6J mice, which underwent sleep studies after vehicle (saline) and clozapine-N-oxide (CNO) treatment.

**Phenotype**	**N**	**Age (weeks)**	**Body weight (g)**	**Body temperature (°C)**
Vehicle	8	19.1 ± 1.5	28.1 ± 1.3	35.1 ± 0.1
CNO	8	20.4 ± 1.6	28.4 ± 1.4	35.2 ± 0.1

*Values are presented as means ± SEM*.

**Table 2:** Sleep architecture in lean and diet-induced obese C57BL/6J mice.

**Phenotype**	**Sleep efficiency(% total time)**	**Sleep duration (min)**			**Sleep bouts**			
					**Number**		**Length (min)**	
		**Total**	**NREM**	**REM**	**NREM**	**REM**	**NREM**	**REM**
Vehicle	45.1 ± 5.1	172 ± 20	166 ± 18	7.3 ± 2.9	26 ± 4	12 ± 2	2.5 ± 0.4	1.1 ± 0.2
CNO	41.0 ± 3.7	138 ± 10	127 ± 9	12.1 ± 1.8	21 ± 4	14 ± 2	2.1 ± 0.4	1.3 ± 0.1

*Values are presented as means ± SEM*.

*CNO, clozapine-N-oxide*.

**Table 3:** Physiology of non-flow limited breathing in C57BL/6J mice, which underwent sleep studies after vehicle (saline) and clozapine-N-oxide (CNO) treatment.

	**V**_**E**_		**Vt**		**RR**		**SPO**_**2**_	
	**NREM**	**REM**	**NREM**	**REM**	**NREM**	**REM**	**NREM**	**REM**
Vehicle	36.5 ± 11.1	34.5 ± 12.2	0.21 ± 0.04	0.16 ± 0.01	178 ± 64	237 ± 121	94.4 ± 2.4	94.3 ± 4.2
CNO	38.5 ± 14.3	31.6 ± 13.1	0.27 ± 0.09	0.16 ± 0.07	147 ± 33	218 ± 91	95.0 ± 0.8	92.1 ± 3.8

*VE, minute ventilation; Vt, tidal volume; RR, respiratory rate, SpO2, mean oxyhemoglobin saturation Values are presented as means ± SEM*.

**Figure 5:**
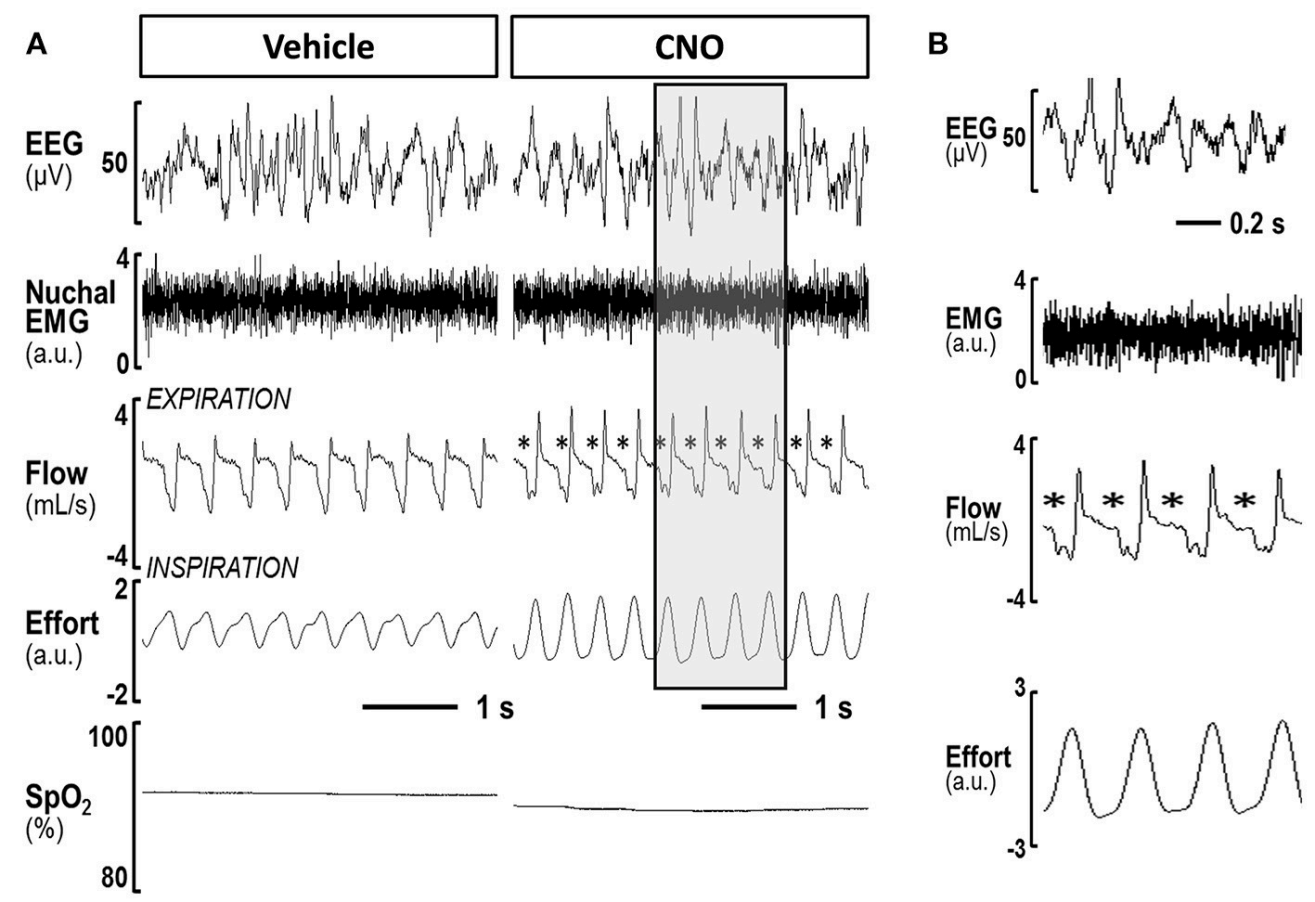
**(A)** A representative trace of NREM sleep after saline and after CNO delivery in lean mice. Panels shows compressed recording of EEG, nuchal EMG, respiratory flow, effort and pulse oximetry (SpO2); The asterisks indicate flow limited breaths; **(B)** The shaded area on the right panel is decompressed.

**Figure 6:**
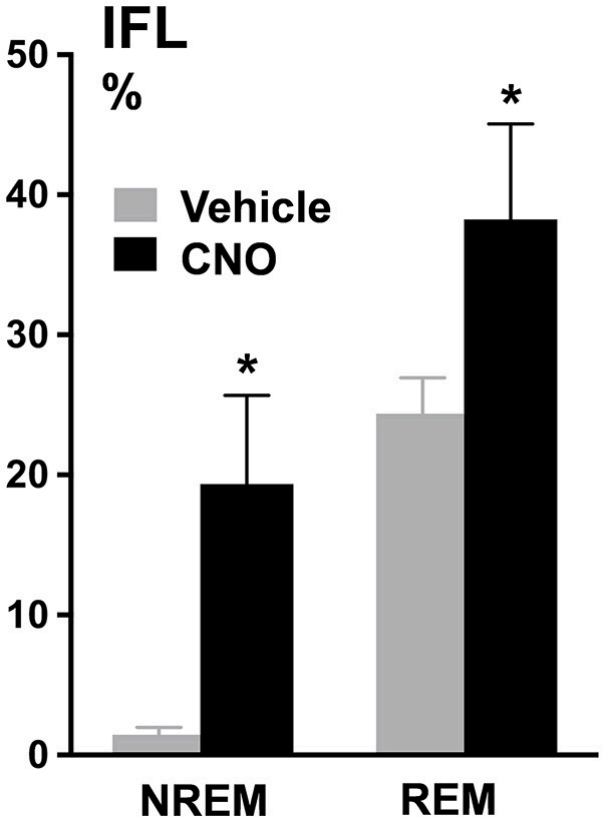
Percentage of flow limited breaths in both NREM sleep (1.9% ± 1.3% baseline vs. 14.2% ± 3.4% CNO, *p* < 0.05) and REM sleep (22.3% ± 4.1% baseline vs. 30.9% ± 4.6% CNO, *p* < 0.05). ^*^p < 0.05.

In contrast, inhibiting hypoglossal motoneuron activity with CNO induced a dramatic increase in the frequency of flow limited breaths expressed as the percentage of all breaths (Figures 5, 6). IFL frequency increased both in NREM sleep (from 1.9 ± 1.3% after vehicle injection to 14.2 ± 3.4% after CNO, *p* < 0.05) and REM sleep (from 22.3 ± 4.1% to 30.9 ± 4.6%, respectively, *p* < 0.05; Figure 4). CNO did not affect minute ventilation or Vimax during flow limited breathing (Table 4). There was no change in the mean SpO_2_, ODI or T90.

**Table 4:** Physiology of inspiratory flow limited breathing in C57BL/6J mice, which underwent sleep studies after vehicle (saline) and clozapine-N-oxide (CNO) treatment.

	**V**_**imax**_		**V**_**E**_		**Vt**		**SPO**_**2**_		**ODI4**		**T90**
	**NREM**	**REM**	**NREM**	**REM**	**NREM**	**REM**	**NREM**	**REM**	**NREM**	**REM**	
Vehicle	2.5 ± 1.4	2.3 ± 0.7	33.7 ± 16.9	34.7 ± 23.1	0.17 ± 0.3	0.1 ± 0.0	93.9 ± 3.5	94.5 ± 4.3	3.5 ± 1.1	18.7 ± 9.0	12.3 ± 2.3
CNO	3.2 ± 0.9	1.9 ± 0.7	49.0 ± 16.8	30.1 ± 10.3	0.24 ± 0.3	0.1 ± 0.0	94.9 ± 3.3	91.4 ± 3.7	1.4 ± 0.5	11.4 ± 4.2	19.1 ± 8.6

*Vimax, maximal inspiratory flow; V_E_, minute ventilation; Vt, tidal volume; SpO_2_, mean oxyhemoglobin saturation; ODI4, oxygen desaturation index (≥4% from baseline) per hour of sleep; T90, the percentage of total sleep time spent with SpO_2_ <90%. Values are presented as means ± SEM*.

## Discussion

To our knowledge, this study demonstrates that effective silencing of hypoglossal motoneurons with chemogenetic techniques leads to pharyngeal airflow obstruction during *natural* sleep in mice that closely mimics sleep disordered breathing in humans. We confirmed by single cell recording that CNO silenced hypoglossal motoneurons containing inhibitory DREADDs and performed polysomnographic recordings in lean wildtype mice expressing inhibitory DREADDs in the hypoglossal nucleus. The main finding of the study was that, in the absence of any predisposing anatomical factors, silencing of hypoglossal motoneurons led to partial upper airway obstruction during sleep, but recurrent hypopneas and apneas were absent. This pattern of sleep disordered breathing is similar to snoring and obstructive hypoventilation in children (38, 39). Our findings are consistent with a current understanding of the pathogenesis of OSA suggesting that, in addition to neuronal dysfunction, compromised pharyngeal anatomy should be present for frank OSA to develop. In discussion below we will further elaborate on our findings in the context of the pathogenesis of OSA and development of animal models.

The anatomic predisposition and elevated mechanical load on the upper airway imposed by obesity play a key role of the pathogenesis of OSA (14, 40–43). However, robust dynamic neuromuscular response protects airway patency and may prevent OSA (44), and *vice versa*, increased mechanical loads and blunted neuromuscular responses are both required for the development of OSA (14). Based on these findings we have previously formulated *a two hit* hypothesis of the OSA pathogenesis (14, 24). Nevertheless, it remained unknown whether diminished neuromuscular response alone without compromised anatomy can lead to sleep disordered breathing and OSA.

Our lab performed extensive studies of upper airway physiology in mice and demonstrated that lean wildtype mice lack anatomical predisposition to OSA (25, 29). In contrast, obese rodents have extensive fat depositions and increased mechanical load on the upper airway, similar to obese humans (25, 29, 41, 45). We have also performed full polysomnographic recordings in genetically obese *ob/ob* mice (26, 46) and C57BL/6J mice with diet induced obesity (23). The main focus of these studies was to examine the prevalence and severity of IFL, a defining feature of pharyngeal obstruction during sleep. We have shown that obesity leads to IFL during both NREM and REM sleep. In contrast, lean mice had occasional flow limitations during REM sleep, but no evidence of IFL during NREM sleep (23). In the current paper, we demonstrated that inhibition of hypoglossal motoneurons induces inspiratory flow limitation in lean mice. The genioglossus muscle of the tongue is the main pharyngeal dilator innervated by the hypoglossal nerve (47–49). An increase in genioglossus activity has a protective effect in OSA (50–53). Although current technology does not allow exclusive targeting of genioglossus as opposed to other muscles of the tongue, we targeted DREADD injections at the ventral rostral portion of the 12 nucleus, which is enriched by motoneurons innervating the upper airway dilator (35, 36). We provide the first evidence that silencing of hypoglossal motoneurons and lingual muscles alone (without other pharyngeal muscles) is sufficient to cause pharyngeal obstruction during NREM sleep in the absence of anatomic predisposition to sleep disordered breathing.

The main characteristic of OSA is recurrent airway obstruction during sleep, which is measured by the apnea hypopnea index or by oxygen desaturation index (ODI). *ob/ob* mice show significant increase in the ODI, especially during REM sleep (30). In contrast, mice with diet induced obesity did not show any increase in ODI, compared to lean mice with the stable airway (23). Similarly, in our present study, inspiratory flow limitation did not result in frank OSA and hypoxia indexes did not increase in inhibitory DREADD-treated mice after CNO administration. These findings imply that uncompromised animals are capable to defend their respiratory function even when hypoglossal neurons are inhibited. In fact, mice preserved their maximal inspiratory flow and minute ventilation during obstructed breaths, which may suggest activation of other pharyngeal muscles to compensate for genioglossus deficiency. It is conceivable that inspiratory flow limitation increased work of breathing generating higher CO_2_ levels, which activated protective neuromuscular responses during sleep (54). Sleep disordered breathing in children frequently has similar features demonstrating persistent IFL without typical OSA (55, 56). In addition to compromised anatomy and neuromuscular responses, OSA cycling is predicated on other factors such as overly robust ventilatory control and low arousal threshold (57–60), which may not be perturbed in our mouse model or in children without OSA. Thus, although silencing of hypoglossal motoneurons predisposes to sleep disordered breathing, other risk factors, such as compromised pharyngeal anatomy, may be necessary to breach compensatory mechanisms preventing the development of frank OSA.

Our study had several limitations. First, we used non-Cre-dependent DREADDs, which could be expressed non-selectively in functionally distinct cell populations. The DREADD had a neuron-specific synapsin driver and therefore the expression was limited to neurons. DREADDs were administered rostrally, but not caudally to the obex, in a portion of the 12th nucleus containing motoneurons innervating tongue protrudors (61). Moreover, experiments with injection of CTB confirmed that DREADDs were predominantly localized in hypoglossal motoneurons innervating the genioglossus muscle, the main pharyngeal dilator. Nevertheless, hypoglossal motoneurons innervating lingual retractors and intrinsic muscles of the tongue as well as respiratory control neurons could be involved. In fact, a trend to deeper and slower respiration observed during non-flow limited breathing after CNO injections (Table 3) may be related to these off-target effects on ventilatory control. Second, we did not examine the effect of chemogenetic inhibition in obese mice. However, the goal of the study was to determine whether silencing of the hypoglossal neurons will induce sleep disordered breathing in the absence of other predisposing factors such as obesity. Third, we did not examine the effect of inhibitory DREADDs in female mice. Fourth, persistent IFL could lead to hypoventilation without OSA with CO_2_ elevation as frequently observed in obese children (62). However, we do not have technical ability to continuously measure CO_2_ levels in mice during sleep. Finally, it has been recently shown that CNO is not entirely selective and, in fact, can be metabolized to clozapine. However, we used a very low dose of CNO, and therefore, its effect was probably specific (63).

In conclusion, we have shown that chemogenetic silencing of the hypoglossal motoneurons in lean mice leads to IFL, which is similar to that observed in humans with sleep disordered breathing, especially in asymptomatic snorers and children with obstructive hypoventilation without recurrent breathing events characteristic of adult OSA. We propose that silencing of the hypoglossal neurons will lead to overt OSA when this neurologic deficit is combined with obesity or craniofacial abnormalities. The rodent model will likely lend itself to studies examining the impact of other pathogenic factors on disease expression, as well as the effects of OSA on cardiometabolic outcomes.

## Ethics statement

This study was carried out in accordance with the recommendations of Johns Hopkins University Animal Care and Use and by George Washington University Animal Care and Use. The protocol was registered and approved by Institutional Animal Care and Use Committee (IACUC) of both Universities.

## Author contributions

TF: study design, experiments, data collection, and writing; HP, RL, and AA: data collection; OD and SB: experiments and data collection; CF: experiments; LS: writing; DM: experiments and writing; AS and VP: study design and writing.

### Conflict of interest statement

The authors declare that the research was conducted in the absence of any commercial or financial relationships that could be construed as a potential conflict of interest.
